# Potential Harmful Effects of PM_2.5_ on Occurrence and Progression of Acute Coronary Syndrome: Epidemiology, Mechanisms, and Prevention Measures

**DOI:** 10.3390/ijerph13080748

**Published:** 2016-07-25

**Authors:** Xu Meng, Ying Zhang, Kun-Qi Yang, Yan-Kun Yang, Xian-Liang Zhou

**Affiliations:** Department of Cardiology, Fuwai Hospital, National Center for Cardiovascular Disease, Chinese Academy of Medical Sciences and Peking Union Medical College, No. 167, Beilishi Road, Beijing 100037, China; mengxu1219@hotmail.com (X.M.); zhangyingfuwai@163.com (Y.Z.); kunqiyang@126.com (K.-Q.Y.); yangyankun66@126.com (Y.-K.Y.)

**Keywords:** acute coronary syndrome, PM_2.5_, epidemiology, mechanisms, prevention measures

## Abstract

The harmful effects of particulate matter with an aerodynamic diameter of <2.5 µm (PM_2.5_) and its association with acute coronary syndrome (ACS) has gained increased attention in recent years. Significant associations between PM_2.5_ and ACS have been found in most studies, although sometimes only observed in specific subgroups. PM_2.5_-induced detrimental effects and ACS arise through multiple mechanisms, including endothelial injury, an enhanced inflammatory response, oxidative stress, autonomic dysfunction, and mitochondria damage as well as genotoxic effects. These effects can lead to a series of physiopathological changes including coronary artery atherosclerosis, hypertension, an imbalance between energy supply and demand to heart tissue, and a systemic hypercoagulable state. Effective strategies to prevent the harmful effects of PM_2.5_ include reducing pollution sources of PM_2.5_ and population exposure to PM_2.5_, and governments and organizations publicizing the harmful effects of PM_2.5_ and establishing air quality standards for PM_2.5_. PM_2.5_ exposure is a significant risk factor for ACS, and effective strategies with which to prevent both susceptible and healthy populations from an increased risk for ACS have important clinical significance in the prevention and treatment of ACS.

## 1. Introduction

The Great Smog event in London [1] resulted in an estimated death toll in excess of 4000 people because of the significant short-term increase in the concentration of particles in ambient air. The event was effectively controlled, but researches on the physiological effects induced by the event are ongoing. At present, atmospheric contamination is a serious problem in developing countries such as China. The Global Burden of Diseases, Injuries, and Risk Factors Study 2010 indicated that particulate matter with an aerodynamic diameter (AD) of <2.5 µm (PM_2.5_) induced 1.2 million early deaths and 25 million disability-adjusted life-years in China in 2010.

In recent decades, PM_2.5_, which can directly enter the alveoli of the lungs, has gained substantial attention worldwide because of its specific aerodynamic and toxicological properties and potentially disastrous effects on human health. Originally, research mainly focused on the adverse impact of PM_2.5_ on the respiratory tract. Over time, a large body of epidemiological research has demonstrated that cardiovascular diseases, especially acute coronary syndrome (ACS), are also closely related to PM_2.5_.

Cardiovascular diseases are a leading cause of death and disability worldwide, and ACS deserves special attention as one of the main causes of cardiovascular death [2]. A substantial number of studies have focused on whether and how elevated PM_2.5_ exposure impact ACS, reaching increasingly more consistent and accurate conclusions. Overall, the majority of research suggests that PM_2.5_ induces an increased risk for ACS [3], although this finding is sometimes only evident in specific subgroups [4]. Elucidation of the relationship between ambient atmospheric PM_2.5_ and ACS and the potential mechanisms may provide new methods with which to prevent people from developing ischemic cardiovascular disease and reduce the incidence of ACS, and even ACS-related death.

## 2. Fundamental Presentation of PM_2.5_ and ACS

### 2.1. Main Sources and Pathogenic Properties of PM_2.5_

Particulate matter is mainly derived from natural phenomena and human activities. For instance, wildfires, volcanoes, and land dust are classified as natural phenomena. Human-generated sources have been confirmed as the main contributors [5] and are mainly attributable to combustion of fossil fuels, emissions from industrial production, biomass burning, and vehicle exhaust [6]. Most of the toxic heavy metals in the air, including chromium, lead, arsenic, cadmium, and nickel, are important sources of fine particles [7]. Additionally, natural combustion processes are far from efficient, and products of combustion mainly include organic carbon species (such as polyaromatic hydrocarbons and dioxins) and trace metals (e.g., lead and arsenic), which are toxic substances that remain in the ambient atmosphere for days, weeks, or even longer [8].

The pathogenic properties of particulate matter are determined by the size, chemical composition, solubility, geography, source, and various other factors associated with the particulate matter in question [9]. Especially of concern are particle size and composition.

Particulate matter varies in size and can be divided into four categories according to its AD: total suspended particulate (AD < 100 μm); PM_10_ (AD < 10 µm); PM_2.5_ (AD < 2.5 µm); and PM_0.1_ (AD < 0.1 µm). PM_10_, PM_2.5_, and PM_0.1_ are also termed thoracic particles, fine particles, and ultrafine particles, respectively. These categories are generally used as essential indicators of ambient air quality management because of their known effects on human health. Differences in size can lead to different health effects. PM_10–2.5_ particles tend to deposit in the upper trachea and bronchus, while PM_2.5_ particles preferentially reach the end-expiratory airways and alveoli, subsequently inducing a series of physiological and pathological reactions. Incremental accumulation of inflammatory cells and secretion of fibrinogen on the surface of alveoli are enhanced by PM_2.5_, inducing prominent local pulmonary inflammatory responses. The finest components, particularly ultrafine particles, can translocate into the blood circulation and cells, directly injuring endothelial cells and various hemocytes and leading to systemic inflammatory responses [10].

The composition of particulate matter is another critical factor to human health. PM_2.5_ has the highest ability to absorb toxic substances such as heavy metals and microorganisms. Solid and liquid particles adhering to the surface of PM_2.5_ are traditionally classified as organic-rich fractions, which may be more closely associated with cardiovascular disease, and inorganic-rich fractions, which may more readily cause pulmonary disorders [11]. A recent study in China reported that fluffy soot particles play a key role in enhancing the toxicity of PM_2.5_ because of their high intrinsic adhesive capability to stick to toxic matter with different chemical components, generating more complicated compositions of ambient fine particulate matter [12]. Additionally, the World Health Organization listed PM_2.5_ as the 13th leading cause of mortality worldwide in 2010 and that same year, the American Heart Association clearly stated that PM_2.5_ is a controlled risk factor for cardiovascular events [13]. Thus, research on the health impacts of airborne particulate matter has traditionally focused on PM_2.5_.

### 2.2. Basic Mechanisms of ACS

ACS, as one of the most common types of coronary heart disease, is a clinical syndrome caused by acute ischemia of the myocardium and commonly includes unstable angina, ST-elevation myocardial infarction (STEMI), and non-ST-elevation myocardial infarction (NSTEMI). Additionally, sudden coronary death has been recognized as one type of ACS in a broad sense. The pathological changes always begin with endothelial injury, followed by arterial inflammation, excessive release of growth factors, inflammatory factors, and vasoactive factors, and vascular remodeling led by proliferation of fibrocytes and smooth muscle cells, which induce plaque formation and atherosclerosis [14]. Rupture of vulnerable plaques activates the coagulation cascade, leading to thrombosis and blood flow interruption in coronary arteries [15]. NSTEMI occurs secondary to the imbalance between the demand and supply of blood and is usually accompanied by microthrombosis without complete obstruction of the coronary artery. Unstable angina, classified into NSTE-ACS, is pathophysiologically similar to NSTEMI, but without myocyte death. STEMI most often results from rupture of vulnerable plaques followed by thrombus formation and complete coronary lumen occlusion [15].

## 3. Epidemiological Associations between PM_2.5_ and ACS

### 3.1. Short-Term Effects

#### 3.1.1. Acute Myocardial Infarction

Acute myocardial infarction (AMI), which results from myocardial ischemia, should be highlighted because of its high prevalence globally and a variety of detrimental outcomes such as heart failure, arrhythmia, and even sudden cardiac death. Most previous studies have reported consistent results showing that increases in PM_2.5_ exposure for the several days or weeks prior to an AMI event are associated with an increased risk for AMI [16,17]. Such associations were influenced by AMI subtypes, lag times, and individual characteristics. Commonly, women [18], the elderly [19], and people with diabetes [18,20,21] are more prone to be effected by high PM_2.5_ exposure. It is possible that some negative results, such as that seen in a recent Canadian cohort study [22], may be explained by differences in characteristics of the study population, inadequate sample size, sources of PM_2.5_, and exposure levels including both time and concentration. These various factors of influence should be taken into consideration when evaluating PM_2.5_-induced detrimental effects on ACS.

AMI can be classified into NSTEMI and STEMI according to different electrocardiographic abnormalities. However, which specific subsets of AMI are more strongly associated with PM_2.5_ have been less frequently reported. Gardner et al. [23] designed a case-crossover study of 338 patients with STEMI and 339 patients with NSTEMI and concluded that STEMI, not NSTEMI, is associated with the acute increase of PM_2.5_-induced ACS. Another case-crossover study in China included 1016 patients with STEMI and 1733 patients with NSTEMI and also reported that short-term exposure to PM_2.5_ was positively associated with STEMI but not overall AMI and NSTEMI [19]. These uniform results from two epidemiological studies reasonably indicate that the risk for STEMI may be more closely associated with PM_2.5_. Further research on this topic will be helpful to identify those in the population susceptible to the disease.

#### 3.1.2. Sudden Coronary Death

Sudden coronary death, one of the most acute and severe manifestations of ACS, is the most common cause of sudden cardiac death. The vast majority of research has shown that a high level of PM_2.5_ is associated with an increased risk for out-of-hospital cardiac arrest in people with coronary heart disease. A systematic review by Teng et al. [24] involving eight studies published in February 2013 showed a positive association between short-term ambient particulate exposure and the occurrence of out-of-hospital cardiac arrest. Several other studies have also shown similar results [25,26,27]. However, scientists in Stockholm, Sweden reached a conflicting conclusion; finding no association between morbidity of out-of-hospital cardiac arrest and any size of particulate matter studied, including PM_2.5_ [28]. That may because of the relatively lower particulate matter concentration in Stockholm compared with previously studied cities.

Fatal arrhythmia, such as ventricular fibrillation, is one of the most common direct causes of cardiac death [29]. A meta-analysis reported that short-term exposure to PM_2.5_ was associated with increased arrhythmia hospitalization or mortality, which may be because of PM_2.5_-induced autonomic nervous system dysfunction, increased ability of the blood to coagulate, and inflammation [30].

### 3.2. Long-Term Effects

#### 3.2.1. Elevated Incidence of ACS

There is convincing evidence that the incidence of ACS may rise with increasing concentrations of PM_2.5_ [31,32]. A relevant meta-analysis that acquired data from the ESCAPE project was conducted in 11 prospective cohorts in Europe and observed an 18% increased risk for acute coronary events per 5-µg/m^3^ increase in PM_2.5_ (hazard ratio, 1.18; 95% confidence interval, 1.01–1.39) using the European limit (<25 µg/m^3^) as the analysis range. This finding indicated a possible harmful effect of PM_2.5_ on human health. Notably, disastrous effects can also be observed at a PM_2.5_ level of <15 µg/m^3^, which is lower than the current limit provided by European governments [33].

#### 3.2.2. Promoting Progression of ACS

Long-term exposure to PM_2.5_ may be associated with the progression of both subclinical and clinical atherosclerosis, which contributes to the incidence and development of ACS [34]. A significantly increased coronary calcification ratio [35] and higher growth rate of carotid intima–media thickness [36] are observed with elevated exposure levels to PM_2.5_ in population cohort studies. More recent epidemiological studies further confirmed similar conclusions in both young adults [37] and a common population [37]. Progression in carotid intima–media thickness was observed at so-called low PM_2.5_ exposure levels in an animal model [38]. This suggests that relatively low concentrations of PM_2.5_ may still have a detrimental impact, even when concentrations are below the threshold thought to be safe.

A meta-analysis reached different conclusions on associations between carotid intima–media thickness (positive), arterial calcification (negative), and ankle-brachial index (negative), indicating that disparate arteries may have inconsistent susceptibility to PM_2.5_-induced atherosclerosis [39]. Technical limitations made it difficult to show subclinical atherosclerosis of the coronary artery directly, and whether peripheral artery atherosclerosis plays a predictive role in coronary artery atherosclerosis requires further research.

## 4. Potential Cellular and Molecular Biology Mechanisms

Although recent epidemiological studies have verified approximate associations between PM_2.5_ and ACS, solid evidence of the mechanisms behind associations remain unclear. Combinations of exposure level, components of PM_2.5_, coexistent diseases, and other factors influence the onset and development of ACS. Several acceptable cellular and molecular biology mechanisms are described below.

### 4.1. Pulmonary and Systemic Inflammatory Response

After it is inhaled into the airway, PM_2.5_ deposits on the surface of bronchioles and alveoli and induces both local and systematic inflammatory responses as well as oxidative stress, which have been widely confirmed to promote further pathological progression of coronary atherosclerosis, leading to a more deleterious state and a higher risk for AMI in patients with or without ACS.

Concentrations of airway and circulatory inflammatory biomarkers could convincingly reflect the local and systemic inflammatory states. Elevated plasma concentrations of traditional inflammatory biomarkers such as interleukin-1 [40], interleukin-6 [40,41], granulocyte-macrophage colony-stimulating factor [40], tumor necrosis factor-alpha [41], fibrinogen [42], white blood cell and platelet count [42,43], C-reactive protein [42,43], and pentraxin-related protein 3 [44] after high-level exposure to PM_2.5_ have been confirmed via a series of deep research studies in the past decade [43]. Pentraxin-related protein 3, in particular, was put forward as a new vascular inflammatory biomarker for the prognostic prediction of cardiovascular disease, especially in patients with ACS [45].

Cytokines and chemotactic proteins activate a series of pathological processes such as macrophage recruitment, phagocytosis of oxidized low-density lipoprotein cholesterol, cell apoptosis and debris clearance, and plaque formation [46]. While research has confirmed the existence of the inflammatory response, whether and how intracellular transduction signal pathways are regulated by PM_2.5_-induced changes is uncertain.

### 4.2. Endothelial Dysfunction of the Coronary Artery

Endothelial cells secrete endothelium-derived constriction factors and endothelium-derived relaxation factors and maintain the balance between the two according to complicated regulatory mechanisms. Reactive hyperemia, brachial artery flow-mediated dilation, and reactive hyperemia-peripheral arterial tonometry [47] are sensitive indexes indicating the endothelial function of blood vessels [48]. Decreased reactive hyperemia [47] and reduced brachial artery flow-mediated dilation [49] were observed among healthy participants after a certain level of PM_2.5_ exposure. Endothelial injury is extensively considered to be the initial factor for atherosclerosis [37], triggering and promoting a range of subsequent reactions.

#### 4.2.1. Direct Injury of Cells

Fine particulate matter causes injury to the endothelium of coronary arteries mainly through indirect cytotoxicity caused by inflammatory cytokines and oxidative stress, impairing endothelial vasodilatory functions and fibrinolytic activity [50]. In vitro experiments revealed that the death rate of vascular endothelial cells increased with rising PM_2.5_ concentrations, suggesting vascular endothelial injury as the possible method by which PM_2.5_ contributes to endothelial dysfunction [51].

#### 4.2.2. Nitric Oxide-Related Mechanisms

Previous studies have established that endothelial-derived nitric oxide (NO), synthesized from endothelial nitric oxide synthase, plays a central role in regulating vascular tone and reactive oxygen species (ROS) levels in endothelium. Interference in synthesis and over-clearance [47], and dysfunction of subsequent downstream signaling pathways [52] as well as other factors affecting the bioavailability of NO may lead to abnormity in endothelial-dependent vasodilatation.

Tetrahydrobiopterin (BH4), an endothelial nitric oxide synthase cofactor, is required for the production of NO and is reported to improve endothelial function and prevent the progression of multiple cardiovascular diseases [53]. Cherng et al. [54] observed substantially diminished acetylcholine-triggered vasodilation of coronary arteries in diesel exhaust-exposed rats compared with coronary arteries from air exposed rats (*p* = 0.006). At mean time, supplementation with an inhibitor of NOS and a precursor of BH4 could augment and rescue deficient acetylcholine-triggered vasodilation, respectively, suggesting insufficiency of NOS and BH4 induced impaired NO-related vasodilation. However, such conclusions should be extended to PM_2.5_ with caution as the components of diesel exhaust were not analyzed in that research.

In addition, uncoupling of endothelial nitric oxide synthase was shown to occur in animal experiments, after which only superoxide (O^2−^) rather than NO can be produced, reducing vasodilation possibly through deduction of prostaglandin as well as endothelial-derived hyperpolarizing factor pathways [52].

#### 4.2.3. Endothelin-1 Involved Mechanisms

In contrast with NO, endothelin (ET)-1 is one of the strongest promoters of vasoconstriction in the circulatory system and has a remarkably long-lasting action. Its mode of action is through two different kinds of ET receptors, of different locations and mechanisms; these being ET_A_ receptor and ET_B_ receptor, which have been reviewed by Davenport et al. [55].

Exposure to PM_2.5_ was found to be associated with an elevated level of plasma ET-1 in both animal [41,56] and human research [57]. An increased constrictive response to ET-1 of the coronary arteries might be induced by regulation of ET receptors. Directly, upregulation of both ET receptors [58,59] as well as dysfunction of ET_B_ receptors [55] have been observed in several animal experiments, which might upset the balance for proper vascular tone. Upregulation of ET_B_ receptor was associated with pathophysiological conditions in rat coronary arteries [60], and upregulation of the ET_A_ receptor, which contributes to basal constrictor tone, has been observed in human atherosclerosis studies both in vitro and in vivo [55].

#### 4.2.4. Other Factors

Oxidative stress takes part in NO-mediated endothelial vasomotor function after exposure to PM_2.5_. Incremental increases in ROS would inescapably react with NO through nitrative stress [61], bringing with it excessive consumption of endothelial-derived NO and a diminished NO level, and leading to nitration of special tyrosine protein residuals that impair functional proteins such as various enzymes, ion channel proteins, and transport proteins [61,62]. Oxidized cholesterol derivatives, especially 7-ketocholesterol, also play important roles in endothelial dysfunction [63]. Increased levels of cyclooxygenase-2, elevated inflammatory factors, such as tumor necrosis factor-alpha and interleukin-6, have also been reported to take part in endothelium dysfunction in animal experiments [41].

### 4.3. Oxidative Stress and Nitrative Stress

Elevated plasma ROS production has been observed in healthy male volunteers [61], animals [64], and epithelial cells [65] after increased PM_2.5_ inhalation. ROS production initiates a series of cellular reactions including damage to cellular macromolecules (e.g., functional and constitutive proteins, DNA, and membranes), cytotoxicity, and release of inflammatory cytokines, all of which have been reviewed by Ghio et al. [66]. Recently, more direct evidence suggests that acute histopathological injury to the rat heart is associated with PM_2.5_-induced oxidative stress [67].

Oxidative stress can be evaluated by components of ROS production such as superoxide dismutase, inducible nitric oxide synthase, NO, and malondialdehyde as well as the enzyme activities of Na^+^, K^+^-ATPase, and Ca^2+^-ATPase. Total plasma homocysteine, an independent predictor of cardiovascular risk, is also used as a marker of oxidative stress. An epidemiological investigation reported that polymorphism in the catalase (CAT-rs2300181) gene and hemochromatosis (HFE) gene converted the association between ambient PM_2.5_ and total plasma homocysteine levels [68]. Significant elevation of urinary 8-hydroxy-2’-deoxyguanosine, a marker of oxidative DNA damage, has also been observed after elevated PM_2.5_ exposure [69].

Injured oxidant defense systems can increase oxidative stress-induced adverse effects. Glutathione, which mainly exists in the liver, plays a key role in protecting humans from oxidative stress by conversion between reduced glutathione and oxidized glutathione. Glutathione and the glutathione synthesis gene Gclm were demonstrated to modulate human antioxidant capacity after short-term exposure to PM_2.5_ [70]. Polymorphisms in the glutathione S-transferase genes induced by PM_2.5_ exposure are also frequently observed [71], resulting in damage to the crucial role of glutathione in antioxidant defense. Additional supplementation of glutathione is considered as a potential method to protect susceptible populations from particulate matter-related injuries.

Oxidative stress is always accompanied by nitrative stress. In the nitrative stress reaction, ROS products biochemically react with NO and NO-derived reactive nitrogen species, producing reactive nitrogen species (such as peroxynitrite) and leading to damage to DNA, proteins, and lipids [72]. Elevation of plasma 3-nitrotyrosine, the index reflecting nitrative stress level, was observed by Kumarathasan et al. [73] after inhalation of concentrated particulate matter in animal experiments. Additionally, the decreased NO level via nitrative stress may also indirectly induce atherosclerosis [74]. However, few studies have focused on nitrative stress-related harmful effects induced by PM_2.5_, and further in-depth research is required.

### 4.4. Autonomic Dysfunctions

Normal heart rhythm originates from sinoatrial node cells and is modulated by the cardiac autonomic nervous system. Combinations of PM_2.5_ and various toxins migrating into the circulating blood may cause dysfunction of the cardiac autonomic nervous system, which may disturb the normal electrical activity of the heart and induce secondary tachyarrhythmia, fatal arrhythmia, and cardiovascular events [30,75].

Heart rate variability has been described as a marker of cardiac autonomic dysfunction and a predictor of sudden cardiac death [76]. A meta-analysis [77] found a statistically significant reduction in heart rate variability in both time-domain and frequency-domain measurements with increased short-term exposure to PM_2.5_, especially among patients with basic cardiovascular disease. However, such a detrimental effect is suspiciously time-dependent. Another study found no alteration in heart rate variability after 1-h exposure to a high concentration of diesel exhaust [78], a finding that is consistent with the widely accepted view that such abnormalities are attributable to systemic inflammation and oxidative stress but not immediate damage to the cardiac autonomic nervous system [69]. Lee et al. [69] found reduced nocturnal heart rate variability and an increased heart rate with elevated biomarkers of oxidative stress and systemic inflammation in blood and urine samples from study participants exposed to a relatively high level of PM_2.5_, suggesting epidemiological evidence that excessive oxidative stress and systemic inflammation may lead to worse PM_2.5_-induced cardiac autonomic dysfunction. Animal experiments have provided evidence to support this view [79].

At the gene level, evidence suggests that PM_2.5_-induced polymorphisms in glutathione S-transferase genes [71] and long GT repeats in the heme oxygenase-1 promoter region [80] may impact autonomic control of the heart, leading to decreased heart rate variability with increased PM_2.5_ exposure.

### 4.5. Mitochondrial Damages

Damage to mitochondria in the myocardium is a potential pathway leading to heart injury, as suggested by studies on cells [81], animals [64], and specific populations involving mother-newborn pairs [82]. Li et al. [64] observed morphologic abnormalities in mitochondria (e.g., swelling, cristae disorder, vacuolation) after inhalation of PM_2.5_, which might subsequently affect gas exchange functions and lead to mitochondrial damage. Increased levels of optic atrophy protein 1, mitofusin 1, dynamin-related protein 1, and fission-mediator protein 1, which are used as mRNA and protein expression-specific fission/fusion markers, have also been found in myocardial tissue of rats after different inhalation concentrations of PM_2.5_ [64]. Whether such changes in mitochondrial gene expression have any influence on the physiological function of mitochondria has not been investigated.

Damage to the mitochondrial respiratory chain induced by particulate matter, which may lead to reduced energy supply to heart tissue, was investigated by Marchini et al. [83]. The researchers observed abnormal patterns of mitochondrial respiration, decreased mitochondrial respiratory complex activity (complex II), decreased mitochondrial membrane potential, insufficient adenosine triphosphate production, and subsequent bioenergetic dysfunction using heart tissue cubes from mice after acute particulate matter exposure. Mitochondria also play an important role in ischemic preconditioning by repeatedly releasing small doses of ROS so that specific organs, including the myocardium, can acquire greater tolerance to larger and persistent ischemic injury [84]. Damage to the pre-protective mechanism of myocardium during ischemic injury may potentially exacerbate myocardial damage in ACS.

### 4.6. Genotoxic Effects

There are studies [71,80,83,85,86] that support the hypothesis that PM_2.5_ may be associated with damage at the genetic level. Knuckles and Dreher [87] observed genomic alterations with changes in the transcription factor proteome, which may precede pathological alterations to cardiomyocytes. Scientists in Italy identified modulation of gene expression, including 181 upregulated and 178 downregulated genes, in heart tissue of mice treated with short-term exposure to PM_2.5_ [88].

## 5. Potential Physiopathologic Mechanisms Mediating PM_2.5_-Induced ACS

### 5.1. Coronary Artery Atherosclerosis

PM_2.5_-induced atherosclerosis can be promoted by the following mechanisms. Increased oxidized cholesterol derivatives, such as 7-ketocholesterol [89], have been observed in both acute and chronic exposure to PM_2.5_, mostly mediated by inflammatory reactions and oxidative stress [90]. PM_2.5_ can promote oxysterol phagocytosis of monocytes and macrophages through upregulation of scavenger receptors such as CD36, leading to increased formation of foam cells with subsequent resultant mobilization of foam cells from the circulation to vascular walls via a toll-like receptor 4 mechanism [90]. Oxysterols transferring into coronary arteries can interact with inflammatory cytokines and growth factors secondarily derived from PM_2.5_-induced vascular inflammation and promote atherosclerotic plaque formation. Widely accepted risk factors for coronary atherosclerosis mainly involving abnormities in blood lipid metabolism [91], hypertension [92], and diabetes mellitus [20,21] are reported to be associated with a high level of PM_2.5_ exposure, and may promote the occurrence and progression of coronary artery atherosclerosis.

### 5.2. Elevation of Blood Pressure

As a traditional risk factor for ACS [15], elevated blood pressure is a potential mechanism leading to PM_2.5_-related increased risk for ACS [93]. A recent meta-analysis reported that short-term exposure to PM_2.5_, but not long-term exposure, was positively associated with an increased risk for hypertension [94]. However, more recent cohort studies [95] and animal experiments [96] have demonstrated that long-term exposure to PM_2.5_ is positively associated with elevated blood pressure. Despite these contrary results, on all available evidence, there is little doubt that a certain degree of PM_2.5_ exposure will affect human blood pressure [94,95]. Some possible mechanisms behind the association between PM_2.5_ and elevated blood pressure are: (1) PM_2.5_-induced systemic inflammation and oxidative stress may lead to elevated sympathetic tone and arterial remodeling through multiple biological mediators; (2) PM_2.5_-induced endothelial dysfunction results in an imbalance in vascular homeostasis, followed by increased peripheral resistance; and (3) PM_2.5_-induced autonomic dysfunction [93].

### 5.3. Imbalance between Energy Demand and Supply to Heart Tissue

An inadequate energy supply to heart tissue after exposure to PM_2.5_ may be through several feasible mechanisms: (1) an increased demand for energy could be induced by an increased heart rate and arrhythmia triggered by PM_2.5_ exposure [97]; (2) insufficient blood perfusion to the heart may result from defective vasomotor regulation [98,99] resulting in ineffective diastole from secondary tachyarrhythmia; and (3) acute damage to the mitochondrial respiratory chain will result in reduced production of adenosine triphosphate [83], which serves as a direct source of energy for heart tissue, resulting in an insufficient energy supply to the heart and a diminished threshold for myocardial ischemia [84,100]. The combined effect of increased consumption and an inadequate supply of energy will promote the onset and development of ACS.

### 5.4. Hypercoagulable State

An increased ability of the blood to coagulate will promote the formation of coronary artery thrombosis and plaque rupture, which can lead to coronary obstruction and ACS. Kloog et al. observed a significant increase in hospitalizations for deep vein thrombosis and pulmonary embolism associated with PM_2.5_ exposure, providing epidemiological evidence for a PM_2.5_-induced hypercoagulable state [101]. Rochelle et al. [102] and Hajat et al. [103] found elevated levels of fibrinogen, endogenous thrombin, tissue-plasminogen activator antigen, and plasminogen activator inhibitor type 1 in serum during exposure to high concentrations of PM_2.5_. Particulates in the blood can activate the platelet and blood coagulation systems, increasing the risk for coronary thrombosis. Increased circulative platelet counts [42,43] and increased concentrations of coagulation factors (e.g., fibrinogen [42]) have been observed in study populations, which appears as an inverse effect of a PM_2.5_-related inflammatory reaction. Additionally, the intrinsic blood coagulation pathway could be activated by endothelial injury or particles that directly enter the bloodstream, followed by platelet activation, blood coagulation, and thrombogenesis [104].

## 6. Preventive Measures to Reduce the Detrimental Effects of PM_2.5_

As the potentially noxious effects of PM_2.5_ have become apparent, effective measures should be implemented to keep the PM_2.5_ level below an established safe threshold to protect susceptible populations from an increased risk for ACS.

Reducing the sources of PM_2.5_, such as that from land dust, traffic emissions, electricity generation, cooking, industrial production based on the burning of fossil fuels, and sandstorms is important to decrease the possibility of susceptible populations being exposed to PM_2.5_. By way of an example, research has found that generalizing the use of waste gas-purifying equipment in industrial enterprises and motors [105,106] is an effective method for reducing PM_2.5_ production.

Personal-level interventions may be more important in regions with high levels of ambient particulate matter pollution. Patients with underlying diseases, the elderly, children, and people with more than one cardiovascular risk factor such as hyperlipidemia [91] and diabetes [20,21] are reportedly susceptible to ACS through stimulation by PM_2.5_. Additionally, there is evidence that PM_2.5_ can harm healthy individuals [49,61]. Therefore, it is suggested that both susceptible populations and healthy people minimize their exposure to PM_2.5_. Reducing egression time, keeping windows closed [107], using activated charcoal and air purifiers [108,109], wearing highly effective dustproof masks [110], and keeping clean can effectively reduce exposure to PM_2.5_ and absorption of toxic materials adherent to PM_2.5_ [111]. Sufficient vitamin intake and clean drinking water are helpful to prevent PM_2.5_-induced damage by maintaining the integrity of the skin and reducing oxidative stress [111]. Governments should clearly publicize the harmful effects of PM_2.5_ and inform citizens of the methods through which they can acquire data on local PM_2.5_ levels and proper preventive measures [112].

Establishing air quality standards for PM_2.5_ is also an important measure to decrease the harmful effects of PM_2.5_, a measure that has been adopted by many countries. The US implemented an air quality standard for PM_2.5_ in 1997, and revised it 2006, setting 15 μg/m^3^ and 35 μg/m^3^ as 1-year and 1-day threshold values, respectively [113]. China established an ambient air quality standard for PM_2.5_ in 2012, which was implemented in 2016. Similar air quality standards have also been established in other countries and by other organizations [113].

## 7. Perspectives and Significance

Future research should aim to accurately elucidate the associations between PM_2.5_ and ACS, explore which of several constituents of PM_2.5_ play a leading role in the clinical pathological process of ACS, find suitable PM_2.5_ thresholds to inform policy formulation, establish appropriate prevention measures to protect vulnerable populations, find effective therapies for people who cannot avoid the detrimental effects of PM_2.5_, and identify vulnerable populations and individuals so that more individualized precautionary measures can be implemented. Detailed studies on these points will be helpful to provide consistent conclusions regarding the associations between PM_2.5_ pollution and ACS risk as well as for the formulation of more efficient prevention measures to protect humans, especially susceptible populations, from an increased risk for ACS induced by PM_2.5_.

## 8. Conclusions

Exposure to PM_2.5_ in ambient air is a significant risk factor for morbidity and the progression and prognosis of ACS. Effective strategies are required to prevent both susceptible and healthy populations from the increased risk for ACS from excessive exposure to PM_2.5_. These recommendations have profound clinical significance for the prevention and treatment of ACS.
